# Relationship between bullet diameter and bullet defect diameter in human calvariums

**DOI:** 10.1007/s00414-019-02197-9

**Published:** 2019-11-16

**Authors:** W. Kerkhoff, E. J. A. T. Mattijssen, E. A. Zwanenburg, R. J. Oostra

**Affiliations:** 1 Netherlands Forensic Institute, PO Box 24044, 2490 AA The Hague, The Netherlands; 2 Behavioural Science Institute, Radboud University Nijmegen, PO Box 9104, 6500 HE Nijmegen, The Netherlands; 3 Faculty of Science and Engineering, University of Groningen, PO Box 72, 9700 AB Groningen, The Netherlands; 4 Department of Medical Biology, Amsterdam UMC, Meibergdreef 9, 1105 AZ Amsterdam, The Netherlands

**Keywords:** Bullet defect, Bullet deformation, Human bone, Calvarium, Cranium, Skull

## Abstract

Existing literature on the relationship between bullet diameter and bullet defect diameter in the human calvarium is summarized and discussed. The hypothesis, derived from the literature, that bullet deformation influences bullet defect diameter was studied in a small controlled experiment. The mean defect size caused by non-deforming projectiles was found to be smaller than the mean defect size caused by deforming projectiles of equal original mass and size. The *p* value of the difference between the two means, measured in two different ways, was found to be 0.002 for both in a Mann–Whitney *U* test and was significant if the confidence level is set at 5%.

## Introduction

In a recent shooting incident in The Netherlands, a defense attorney claimed that his client could not have fired the fatal shot, because the size of the bullet defect in the victim’s sternum was measured to be smaller than the caliber of his client’s firearm. In two cases in the USA, similar claims were made regarding the medical examiners’ reported entry defect diameters in skull bone which were slightly smaller than the 9 mm and .45-caliber bullets which produced them. In all three cases, the defense rationale was that, because bone is a hard material, a defect in bone must be equal in size or larger than the bullet that caused it. This idea seems to be supported by Dodd [1] who mentions that the dimension of a bullet defect in bone may be that of the bullet or larger. Other sources [2–4] indicate that bullet defects are usually smaller than the bullets that caused them. Di Maio [2] mentions that bone has some elasticity and that a bullet may produce a defect smaller than itself. This observation is confirmed by Sellier and Kneubuehl [3], referring to Berg [4]. Like Di Maio, Sellier and Kneubuehl mention that this is true for both deforming and undeforming projectiles. Di Maio mentions that a 9-mm bullet may produce an 8.5-mm defect but cannot produce a 7.65-mm defect. The studies by Berryman et al. [5], Ross [6], Paschall and Ross [7], and Kuhl and Janssen [8] focused on bullet defects in human cranial bone and will be discussed in more detail below.

## Literature

### Review

Berryman et al. [5], Ross [6], and Paschall and Ross [7] studied calvarium samples from forensic collections, with defects caused by bullets of a known caliber. The notion of a “caliber” in the three studies was implicitly defined as the nominal bullet diameter, or rather a range of bullets with the same diameter, without reference to specific cartridge designations. The size of each defect was compared to the diameter of the caliber group to which the causative bullet was assigned. The different caliber groups will be referred to as .22, .25, .32, and .38 in this review, following the nomenclature of the three studies. Berrymann et al. [5] measured the maximum diameter size of 16, 8 and 11 defects, known to be caused by .22, .25 and .38 caliber bullets, respectively. The measurements were performed with a caliper on the outer rim of the entrance side of the defects. The mean diameter of the defects caused by the .22, .25 and .38 bullets was measured to be 36%, 12%, and 21% larger than the respective bullet diameters. Some individual measurements in the .38 caliber group where measured to be smaller. This was not the case in the .22 and the .25 caliber group. Ross [6] measured 37, 5, 6, and 25 defects, known to be caused by .22, .25, .32, and .38 caliber bullets. The mean minimum diameters of the defects caused by the .22, .25, .32, and .38 bullets were measured to be 18%, 6%, 14%, and 22% larger than the respective calibers. Some individual defect measurements in the .25, .32, and .38 caliber groups were measured to be smaller than the calibers of those groups. This was not the case in the .22 caliber group. The study by Ross indicated that the defect diameter is influenced by not only the caliber of the bullet but also by the thickness of the bone, in the sense that bullets produce larger defects in thicker bone. Paschall and Ross [7] studied a total of 169 samples, 68 of which were studied by the authors directly and 101 of which were taken from the literature [9]. Of all defects, 77 were caused by .22 caliber bullets, 40 by .32 caliber bullets, and 52 by .38 caliber bullets. The authors tried to classify the samples by their minimum defect size to the known caliber group. The overall misclassification rate was 41%. A study of an eighteen sample sub set indicated that the difference in bullet defect diameter is influenced by not only the caliber of the bullet and bone thickness, but also by bone mineral density (BMD) in the sense that bullets produce larger defects in bone with a higher BMD. The influence of BMD on defect size was found to be larger than that of bone thickness.

Kuhl and Janssen [8] fired 96 bullets orthogonally (90° in all directions) at heads of fresh human remains, still containing a brain, some with and some without overlying soft tissue. The bone around each bullet defect was sawed out, soaked for 14 days in a 4% formalin solution and air-dried. A further 113 bullets were fired orthogonally at fresh human calvariums, 18 of which had the overlying soft tissue removed before shooting. The thickness as well as the BMD of the samples was measured. The authors used cartridges of six different calibers in their study. The calibers used in this study were defined as specific cartridge designations instead of caliber groups, as was the case in the aforementioned studies [5–7]. The used .32 Auto, .380 Auto and 9 mm Luger cartridges were loaded with full metal jacket bullets. The .32 Long Colt, .38 S&W and .38 Special cartridges used were loaded with lead bullets. The bullet defect size was measured with a conical (tapered) shaft, placed in each defect. The diameter of the cone at the position it stuck in the defect was measured. The diameters of the defects were found to be about equal in the samples taken from the heads and taken from the calvariums. The mean diameters of the defects caused by the .32 Auto, .380 Auto, and 9 mm Luger full metal jacket bullets were 6% larger, about equal in size, and 3% smaller than the original bullet diameters, respectively. The mean diameters of the defects caused by the .32 Long Colt and .38 S&W lead bullets were measured to be 5–9% and 8% larger than the original bullet diameter, respectively. The mean diameter of the defects caused by the .38 Special lead bullets fired at heads and unmacerated calvariums were 24% larger than the original bullet diameter. The mean diameter of the defects caused by these bullets in the 18 samples without overlying soft tissue were measured to be only 16% larger than the original bullet diameter. The study by Kuhl and Janssen, as did the study by Ross [6], indicated that bullets have a tendency to produce larger defects in thicker bone and, as found by Paschall and Ross [7], have a tendency to produce larger defects in bone with a higher BMD.

### Comments

The studies by Berrymann et al. [5], Ross [6] and Paschall and Ross [7] were conducted with casework samples, which has some drawbacks over controlled experiments. One relevant factor that cannot be controlled is the angle of attack, also known as the incidence angle of a bullet. In a shooting incident, a bullet will rarely hit a target with a perfectly orthogonal trajectory. A bullet that hits at an angle will produce an elliptical defect. Both Berrymann et al. and Ross selected round defects for their studies, but this appears to have been a visual assessment. A selection for roundness was not explicitly mentioned in de study by Paschall and Ross. If the edge of a defect was not completely sharp and defined, a slightly elliptical shape might not have been recognized as such. If the sample sets did indeed include slightly elliptical defects, this could have introduced a small bias in the reported mean values towards oversized measurements. In the study by Kuhl and Janssen, the incidence angle (orthogonal for all shots) is known. Other aspects that leave some room for interpretation are the age and storage conditions of the used samples. There are several ways to preserve and store bone samples, like fresh freezing, preservation in solutions (non-frozen, cooled, frozen) and/or drying. Todd [10] reported an average of 1.1% of shrinkage in all dimensions in 24 macerated and dried skulls from various individuals. In a later study with 48 macerated and dried skulls from all male individuals, Todd [11] measured a 0.8 to 1.0% of shrinkage in all dimensions. Shrinkage was complete after 4 weeks and was attributed by Todd to dehydration. Kuhl and Jansen used fresh samples that were air-dried for an unknown period. The age and storage conditions of the samples used by Berryman et al., Ross, and Paschall and Ross were not mentioned explicitly in the respective papers.

A bigger influence than incidence angle and bone shrinkage on the size of a defect might be caused by bullet deformation. Bullets that deform upon impact with bone might produce larger defects than non-deforming bullets. Some bullet types are more likely to deform than others. Full metal jacket bullets are less likely to deform and/or deform to a lesser extent when they do, than do lead bullets. Different bullet types (prone or not to deformation) are more common in some cartridge types than in others. Full metal jacket bullets are very common in .25 Auto cartridges but are rare in .22 Long Rifle cartridges and vice versa. This might explain why the mean values of the defects in the .22 caliber groups were more enlarged, when related to the original bullet diameter, (36% and 18%) than the defects in the .25 caliber groups (12% and 6%) in the studies by Berryman et al. [5] and Ross [6]. In the study by Kuhl and Jansen [8] to, bullet deformation might explain why the defects caused by the lead bullets were on average measured to be larger than those caused by the full metal jacket bullets.

Based on these studies, we hypothesized that bullet deformation influences bullet defect diameter. We expected bullet defects to be larger when resulting from deforming bullets than from non-deforming bullets.

## Experiments

The experiments described below were not conducted for the specific purpose of studying the relationship between bullet and bullet defect diameter in bone, but are part of a validation study on synthetic bone simulants. Some of the results of the study, presented below, were used to test our hypothesis that deforming bullets produce larger defects than non-deforming bullets.

### Materials

The experiments were performed with projectiles that were selected to keep their original shape (non-deforming projectiles) and others that were expected to deform on impact with bone (deforming projectiles).

#### Non-deforming projectiles

G20 grade 5.5 mm (nominal size) chrome plated high-carbon steel spheres manufactured according to ISO standard 3290-1:2014 [12] were selected as non-deforming projectiles. The actual mean diameter of these projectiles was measured to be 5.48 mm, based on ten measured specimen. All ten measurements read 5.48 mm, indicating that the diameter fell within the ± 0.005 mm tolerance of the used caliper. ISO standard 3290-1:2014 [12] does not dictate a specific hardness. However, the original intended purpose of these spheres (for instance in ball bearings) lead the authors to assume these projectiles would not deform when perforating human bone.

#### Deforming projectiles

Tin/lead alloy spheres where selected as projectiles intended to deform. Pure lead spheres where considered but not used for this study. Lead bullets fired through bone and retrieved in forensic casework have often lost part of their original weight. Because mass loss (bullet fragmentation) would present an extra variable in this study, a harder material than lead was sought. Pure lead has a Brinell hardness of 4 to 6 HB, while a 70/30% tin/lead alloy has a 12 HB hardness [13]. When cast to an approximate 82/18% tin/lead ratio, the density of the alloy is about equal to that of the used steel spheres. The tin/lead alloy spheres where cast using a .22 buckshot mold. The nominal diameter of the 5.56 mm rough cast spheres was mechanically reduced to the 5.48 mm (.216″) diameter of the steel spheres. After selecting specimen with the same mass as the steel balls (0.679 ± 0.0005 g or 10.48 ± 0.005 grain), the surface of the selected tin/lead spheres was smoothened by rolling them between a glass plate and a block of wood.

#### Target material

Two calvariums of recently deceased male individuals, aged 69 and 73 years, were used for this study. This material was obtained through the body donation program of the Department of Medical Biology of the Amsterdam UMC, at the location Academic Medical Center (AMC) in The Netherlands. The bodies from which the calvariums were taken were donated to science in accordance with Dutch legislation and the regulations of the medical ethical committee of the Amsterdam UMC at the AMC. The bodies of the deceased where partially embalmed prior to the removal of the calvariums. In the embalming process, embalming fluid is pumped through the body’s circulatory system via the femoral artery of the left leg. The calvarium, consisting of mayor parts of the frontal bone, the left and right parietal bones and the occipital bone, was separated from the rest of the body after the first hours of the embalming procedure to remove the brain. At that stage, the body fluids in the diploë have been partly replaced by an unknown quantity of embalming fluid. The embalming fluid contains water, ethanol, glycerin, formaldehyde, methanol and phenol. The different components have different functions. We found no literature about the influence on the mechanical properties of bone for the specific embalming fluid used by the AMC. However, there is literature about the effects of its individual components. Since both calvariums where used within a 14-day period after time of death and embalming, only the studies that mention short term effects of embalming are referred to here. A study by Öhman et al. [14] found that a low formalin (a 37% solution of formaldehyde in water) concentration does not have any effect on the mechanical properties of bone in a fixation period of 4 weeks. Mick et al. [15] found no effect after a 14-day fixation period with either formalin or ethanol. Sellier and Kneubuehl [3] and Kneubuehl et al. [16] referring to Huelke et al. [17, 18] mention no differences in ballistic properties between fresh, untreated bones and embalmed bones.

### Method

As many as possible suitable 4 × 4 cm (1.6 × 1.6″) size locations were selected on the two calvariums. The selected locations were relatively even in the middle and away from the sutures between the frontal, parietal and occipital bones. Six and eight of these samples could be cut out of the calvariums of the 69- and 73-year-old bodies, respectively. The thickness of the center of each sample was measured before shooting with a micrometer, at seven evenly spaced locations in the middle of the sample. The measurements were repeated by a second examiner. The means of the fourteen measurements for each sample are included in Tables 1 and 2 as “bone thickness.” For the experiments, each sample was fixed in a mount that also held a barrel with its muzzle at a 60-cm (24″) distance from the sample. The samples were fixed orthogonally with respect to the heart of the barrel, with the convex side (outside) facing the muzzle. The projectiles were propelled through the barrel by means of compressed air. Velocities were measured with a chronograph in flight between the muzzle and the sample. The size of the defects resulting from the fired projectiles was measured both by using a caliper as in the studies by Berryman et al. [5], Ross [6], and Paschall and Ross [7] and with a cone (tapered shaft), as was used by Kuhl and Janssen [8]. For the “caliper” measurements, the diameter of the defects on the convex side of each sample was measured at five, random selected locations. This was done by two researchers. The mean of the ten measurements was taken as a measure for the diameter of the bullet defect on the convex side. For the “cone” measurements, a 6-g aluminum cone was lowered in the defect from the convex (entry) side until it stuck on its own weight. The diameter of the cone, at the height were it stuck in the defect, was measured. The projectiles where caught in a cotton box behind the sample. The diameter of the retrieved projectiles was measured at five, random selected locations on the enlarged part of the circumference of the projectile (on the deformed projectiles) with a digital caliper. This was done by two researchers. The mean of the resulting ten measurements was taken as a measure for the diameter of the deformed projectile. To check for mass loss, all spheres where weighed before and after shooting.

**Table 1:** Results non-deforming projectiles

Bone sample	Bone thickness	Projectile diameter after	Defect diameter CALIPER	Defect diameter CONE
Frontal bone 69-year-old male	7.8 mm (.31″)	5.48 mm (.216″)	5.35 mm (.211″)	5.22 mm (.206″)
Right parietal bone 69-year-old male	7.8 mm (.31″)	5.48 mm (.216″)	5.18 mm (.204″)	5.04 mm (.198″)
Frontal bone 73-year-old male	6.4 mm (.25″)	5.48 mm (.216″)	5.13 mm (.202″)	5.22 mm (.206″)
Left parietal bone 73-year-old male	7.7 mm (.31″)	5.48 mm (.216″)	5.21 mm (.205″)	5.26 mm (.207″)
Left parietal bone 73-year-old male	8.0 mm (.32″)	5.48 mm (.216″)	5.24 mm (.206″)	5.18 mm (.204″)
Left parietal bone 73-year-old male	8.6 mm (.34″)	5.48 mm (.216″)	5.24 mm (.206″)	5.10 mm (.201″)
Mean values ➔	7.7	5.48 mm (.216″)	5.22 mm (.206″)	5.17 mm (.204″)
Standard deviations ➔	0.7	0	0.07	0.08

**Table 2:** Results deforming projectiles

Bone sample	Bone thickness	Projectile diameter after	Defect diameter CALIPER	Defect diameter CONE
Frontal bone 69-year-old male	7.6 mm (.30″)	6.09 mm (.240″)	5.90 mm (.232″)	5.65 mm (.222″)
Left parietal bone 69-year-old male	8.3 mm (.33″)	6.02 mm (.237″)	5.93 mm (.233″)	5.82 mm (.229″)
Right parietal bone 73-year-old male	6.8 mm (.27″)	6.04 mm (.238″)	5.99 mm (.236″)	5.61 mm (.221″)
Right parietal bone 73-year-old male	7.2 mm (.29″)	5.78 mm (.228″)	5.67 mm (.223″)	5.50 mm (.217″)
Right parietal bone 73-year-old male	7.6 mm (.30″)	5.90 mm (.232″)	5.85 mm (.230″)	5.72 mm (.225″)
Frontal bone 73-year-old male	5.0 mm (.20″)	6.05 mm (.238″)	5.92 mm (.233″)	5.74 mm (.226″)
Mean values ➔	7.1	5.97 mm (.235″)	5.87 mm (.231″)	5.67 mm (.223″)
Standard deviations ➔	1.1	0.11	0.11	0.11

## Results

All projectiles perforated the samples. Two of the six bone samples of the 69-year-old individual shattered or broke to an extent that a reliable assessment of the size of the defect was no longer possible. The other four samples of this individual and the eight samples taken from the 73-year-old individual remained intact and showed the typical, circular perforation on the convex (entry) side in combination with a larger, irregular, beveled defect on the concave (exit) side. The projectile velocities of the 12 remaining projectiles measured between 258 m/s (846 fps) and 286 m/s (938 fps) which are typical velocities for subsonic pistol and revolver projectiles. As expected, none of the steel projectiles deformed or lost mass. All tin/lead alloy projectiles flattened on impact to some degree and lost between 0.5 and 1.8% of their original mass. Tables 1 and 2 show the results of the shots with non-deforming and deforming projectiles, respectively.

Two one-sided Mann–Whitney *U* tests [19] with a significance level of 0.05 were performed to test whether bullet defects of deforming bullets are larger than those of non-deforming bullets. Measured with calipers, the defects produced by deforming projectiles were significantly larger (*Mdn* = 5.91) than those produced by non-deforming projectiles (*Mdn* = 5.23), *U* = 0.00, *z* = 2.88, *p* = 0.002, *r* = 0.83. This was also the case when the same defects were measured with a cone. Measured this way, the defects produced by deforming projectiles were also significantly larger (*Mdn* = 5.67) than those produced by non-deforming projectiles (*Mdn* = 5.17), *U* = 0.00, *z* = 2.88, *p* = 0.002, *r* = 0.83.

## Discussion

Defects in the studied literature, caused by bullets that are more likely to deform, appear to produce larger defects in human calvariums than those caused by bullets that are less likely to deform. The results of the current study support the hypothesis that deforming bullets produce larger defects than do non-deforming bullets. Defects produced by deforming projectiles in the current study were measured to be significantly larger than those produced by non-deforming projectiles. The differences cannot be attributed to a difference in bone thickness or a difference in BMD in the sample sets. The mean thickness of the bone samples (see Tables 1 and 2) used for the non-deforming projectiles (7.7 mm, .303″) was more than the mean thickness of the samples used for the deforming projectiles (7.1 mm, .280″). If bone thickness played a role in defect size, the literature [6, 7] indicates that the defects caused by the non-deforming projectiles should have been larger than those caused by the deforming projectiles, because of the thinner bone in the latter sample set. The opposite result was obtained. BMD was not measured for the bone used in the current study. The samples of the two calvariums were equally distributed over the two sets, preventing a possible bias caused by differences in BMD between the two individuals.

Bullet deformation might (partially) be the indirect cause of the fact that defects have a tendency to become larger in thicker bone and/or in bone with a higher BMD, as was reported in the literature [6, 7]. Both thicker bone and bone with a higher BMD offer greater ballistic resistance to a bullet and might cause a bullet to deform more.

## Conclusion and recommendations

Our results support the hypothesis that deforming bullets produce larger defects than do non-deforming bullets and therefor that the size of a bullet defect is dependent on the deforming or non-deforming characteristics of the bullet that produced it. As a consequence, a bullet defect diameter cannot be reliably related to a bullets diameter without taking the possibility of bullet deformation into account. Therefore, an assessment of the used bullet type (deforming or not) must be made, e.g., by examining retrieved bullet fragments or cartridge cases.
